# NFBTA: A Potent Cytotoxic Agent against Glioblastoma

**DOI:** 10.3390/molecules24132411

**Published:** 2019-06-29

**Authors:** Hasan Turkez, Flávio Rogério da Nóbrega, Ozlem Ozdemir, Carlos da Silva Maia Bezerra Filho, Reinaldo Nóbrega de Almeida, Eduardo Tejera, Yunierkis Perez-Castillo, Damião Pergentino de Sousa

**Affiliations:** 1 Department of Molecular Biology and Genetics, Erzurum Technical University, Erzurum 25240, Turkey; 2 Department of Pharmacy, “G. d’Annunzio” University of Chieti-Pescara, Via dei Vestini 31, 66013 Chieti Scalo, Italy; 3 Department of Pharmaceutical Sciences, Federal University of Paraíba, João Pessoa, PB 58051-085, Brazil; 4 Department of Physiology, Federal University of Paraíba, João Pessoa, PB 58051-085, Brazil; 5 Escuela de Ciencias Físicas y Matemáticas, Universidad de Las Américas, Quito 170125, Ecuador

**Keywords:** piplartine, anticancer, analogue, antiglioblastoma therapy, *Piper*

## Abstract

Piplartine (PPL), also known as piperlongumine, is a biologically active alkaloid extracted from the *Piper* genus which has been found to have highly effective anticancer activity against several tumor cell lines. This study investigates in detail the antitumoral potential of a PPL analogue; (*E*)-N-(4-fluorobenzyl)-3-(3,4,5-trimethoxyphenyl) acrylamide (NFBTA). The anticancer potential of NFBTA on the glioblastoma multiforme (GBM) cell line (U87MG) was determined by 3-(4,5-dimethyl-2-thia-zolyl)-2, 5-diphenyl-2H-tetrazolium bromide (MTT), and lactate dehydrogenase (LDH) release analysis, and the selectivity index (SI) was calculated. To detect cell apoptosis, fluorescent staining via flow cytometry and Hoechst 33258 staining were performed. Oxidative alterations were assessed via colorimetric measurement methods. Alterations in expressions of key genes related to carcinogenesis were determined. Additionally, in terms of NFBTA cytotoxic, oxidative, and genotoxic damage potential, the biosafety of this novel agent was evaluated in cultured human whole blood cells. Cell viability analyses revealed that NFBTA exhibited strong cytotoxic activity in cultured U87MG cells, with high selectivity and inhibitory activity in apoptotic processes, as well as potential for altering the principal molecular genetic responses in U87MG cell growth. Molecular docking studies strongly suggested a plausible anti-proliferative mechanism for NBFTA. The results of the experimental in vitro human glioblastoma model and computational approach revealed promising cytotoxic activity for NFBTA, helping to orient further studies evaluating its antitumor profile for safe and effective therapeutic applications.

## 1. Introduction

Glioblastoma multiforme (GBM), the most aggressive type of glioma, is classified by the World Health Organization (WHO) as a grade IV brain tumor associated with high mortality [1,2,3,4]. Despite cancer therapies, including surgery, radiotherapy, and chemotherapy, the median survival with current therapeutic options is only 14.6 months. Great efforts by several laboratories and clinics have increased this survival time by barely 2.5 months. Current treatment strategies for curing malignant gliomas, including GBM, are ineffective. With such poor outcomes, there is a great need for new and innovative therapeutic approaches to GBM [5,6,7]. Yet, recent reports indicate that investigations in GBM at the gene level are rare. The number of gene expression studies involving genetic alterations associated with GBM in cell proliferation regulation, DNA repair, apoptosis, and signal transduction is limited. Thus, more detailed investigations are required to develop novel diagnosis and treatment strategies, which are needed for GBM [8,9]. 

Piplartine (PPL), also known as piperlongumine, is an alkamide found in the genus, *Piper;* it possesses a wide spectrum of biological properties, including anticancer activities against glioblastoma, lymphoma, melanomas, gastric cancer, lung cancer, myeloid leukemia, colon cancer, breast cancer, and prostate cancers as well [10,11,12,13,14,15,16,17,18]. In a study on glioblastoma, it was reported that PPL selectively killed GBM cells via accumulating reactive oxygen species (ROS) to activate c-Jun N-terminal kinase (JNK) and p38 mitogen-activated protein kinase (MAPK) cascades [12]. In addition, PPL inhibited the migration of human glioma (LN229 or U87 MG) cells [19]. This result indicates the therapeutic potential of the compound in suppressing tumor invasion and metastasis. Therefore, these data highlight PPL as a drug candidate against various types of tumors. In fact, other studies show that PPL alters the generation of ROS, erasing cancerous cells while helping to maintain healthy cell growth. Interestingly, PPL is thought to generate endoplasmic reticulum stress, resulting in protein dyshomeostasis and DNA damage in high-grade glioma (HGG) cells. PPL leads to the enhancement of intracellular ROS levels by inactivating an ROS-degrading enzyme, peroxiredoxin 4 (PRDX4), which is overexpressed in most GBM [20]. Previous reports indicate that PPL also has functions at the molecular level and evokes apoptotic cell death through the alteration of differing pathways, such as phosphatidylinositol 3-kinase (PI3K)/serine/threonine protein kinase (Akt) and mammalian target of rapamycin (mTOR) (PI3K/Akt/mTOR), nuclear factor kappa B (NF-κB), Janus kinase-signal transducer and activator of transcription-3 (JAK1, 2/STAT3), and JNK, in cancer-derived cell lines [21]. Regarding the synthetic derivatives of PPL, there are reports of bioactivity against two different human tumor cell lines, human neuroblastoma (IMR-32) and cervical cancer (HeLa) cells [22], while in a study on a PPL analog, the ester, (*E*)-benzhydryl 3-(3,4,5-trimethoxyphenyl) acrylate, was shown to be cytotoxic against U87MG cell line [23].

**Figure 1:**
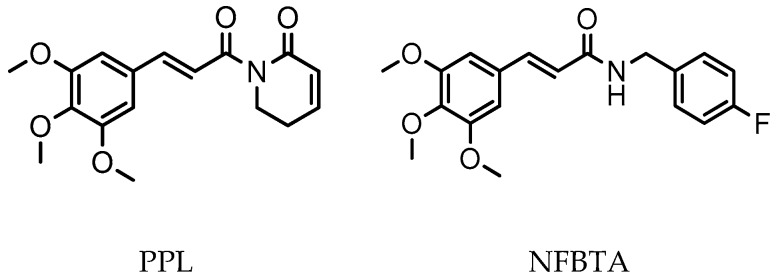
Chemical structures of PPL and NFBTA.

Studies demonstrate that the introduction of halogens and aromatic groups can increase lipophilicity and thus the efficacy of bioactive molecules, as well as their mode of interaction with receptors [24,25]. In a previous study, we explored novel synthesized PPL analogues, having a clear concentration-dependent antiproliferative effect against U87MG cell proliferation via the induction of apoptotic and oxidative processes [23]. We thus planned a new synthesis, (*E*)-N-(4-fluorobenzyl)-3-(3,4,5-trimethoxyphenyl)acrylamide (NFBTA), as a possible lead compound to introduce a novel PPL analogue for testing in pharmacological evaluations. NFBTA is an amide (structurally similar), yet is more lipophilic than PPL (Figure 1), that should penetrate the central nervous system (CNS) more efficiently. This analogue is expected to have better anticancer activity against U87MG glioblastoma cell proliferation and is widely used experimentally for in vitro glioma drug development modeling. The U87MG cell line is the most widely studied cell line as an in vitro human glioblastoma (GBM) model based on its superior features as compared to other lines. In that, proteins for glioma invasion are upregulated more specifically in these cells as compared to U343MG-A. U87MG shows a great tendency to form neurosphere-like structures for nuclei staining to verify proliferation rates. Again, its genome has been fully sequenced in recent studies [26,27,28]. Paclitaxel (PTX) has exhibited great potential in the treatment of different human cancers, including ovarian breast, lung, neck, and colorectal cancers as well as brain tumors. In fact, PTX has been introduced as a new class of microtubule stabilizing agents with excellent antiproliferative action against the growth of glioma. Nominately, PTX was shown to induce apoptotic cell death in human glioblastoma U87MG cells [29,30,31]. Therefore, we selected PTX as a positive control agent for the comparison of anti-glioma activity of NFBTA. The anticancer potential of NFBTA on U87MG cells was assessed using 3-(4,5-Dimethylthiazol-2-yl)-2,5-diphenyltetrazolium bromide (MTT) and lactate dehydrogenase (LDH) release assays, and its apoptotic effects via fluorescent and Hoechst 33,258 staining procedures. The selectivity index (SI) was calculated using cancer cell the half maximal inhibitory concentration (IC_50_) values as compared to human pulmonary alveolar epithelial (HPAEpiC) cells. To elucidate oxidative alterations, total antioxidant capacity (TAC) and total oxidant status (TOS) levels were determined via colorimetric measurement methods. Expression profiling of certain cancer-associated genes was performed by mini-microarray analysis, and biosafety assessments involving cytotoxic, oxidative, and genotoxic damage potentials were evaluated for NFBTA with cultured human blood cells. In addition, molecular docking studies on several therapeutic targets were performed. 

## 2. Results

### 2.1. Anti-Proliferative and Pro-Apoptotic Effect of NFBTA

**Figure 2:**
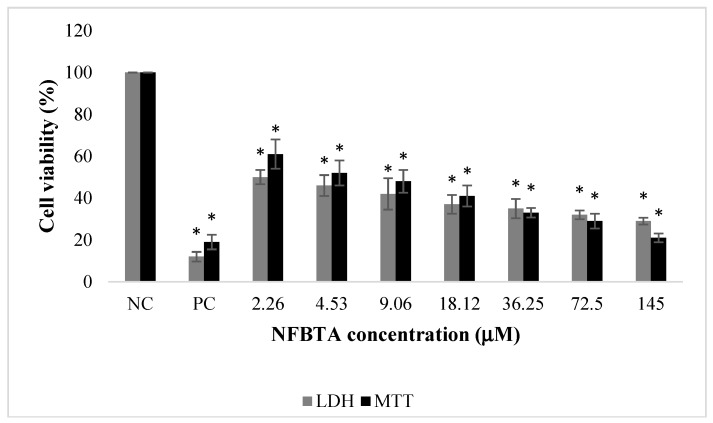
The effects of NFBTA applications (2.26–145 µM) on human glioblastoma U87MG cell proliferation for 48 h. Cell viability was determined using MTT and LDH assays. Data are expressed as the mean ± SD of four repetitive experiments. Statistical analysis was carried out by ANOVA and Duncan’s test. (*) symbol presents statistical differences between NFBTA treated and untreated (NC) cultures at a significance level of *p* < 0.05. Paclitaxel was used as a positive control (PC).

**Figure 3:**
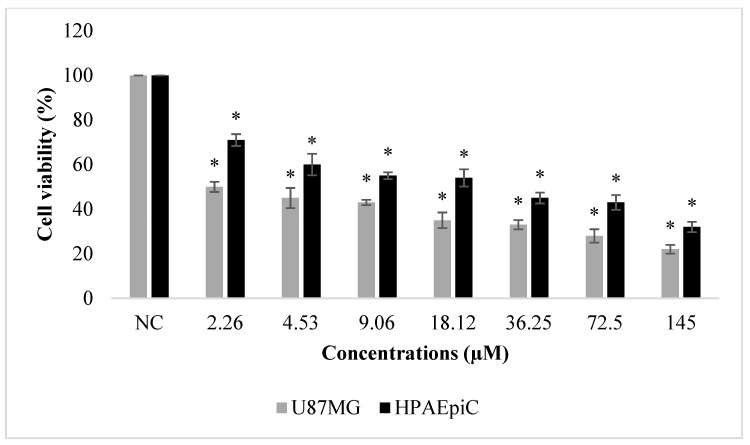
Effects of paclitaxel (2.26–145 µM) on human glioblastoma U87MG and normal human lung cell (HPAEpiC) proliferations for 48 h. Cell viability was determined using the MTT assay. Data are expressed as the mean ± SD of four repetitive experiments. Statistical analysis was carried out by ANOVA and Duncan’s test. (*) symbol presents statistical differences between NFBTA treated and untreated (NC) cultures at a significance level of *p* < 0.05.

**Figure 4:**
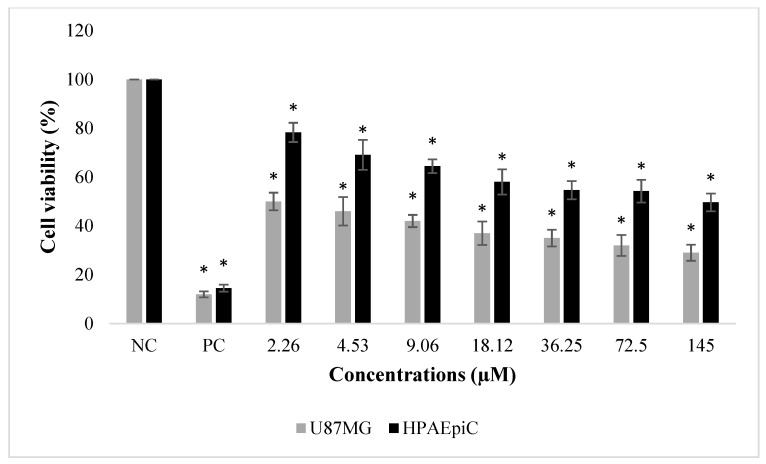
The effects of NFBTA (2.26–145 µM) on human glioblastoma U87MG and normal human lung (HPAEpiC) cell proliferations for 48 h. Cell viability was determined using the MTT assay. Data are expressed as the mean ± SD of four repetitive experiments. Statistical analysis was carried out by ANOVA and Duncan’s test. (*) symbol presents statistical differences between treated (NFBTA) and untreated (NC) cultures at a significance level of *p* < 0.05. Paclitaxel was used as a positive control (PC).

The effects of NFBTA on U87MG cell proliferation were determined by MTT and LDH release analyses. Cells were treated with different NFBTA concentrations (from 2.26 to 145 µM), and the cell viability rates were measured at 48 h after treatment. As shown in Figure 2, both assay results showed that NFBTA (in comparison to the untreated control) significantly (*p* < 0.05) decreased cell viability of the U87MG cell line in a clear concentration-dependent manner. In fact, IC_50_ values for NFBTA and paclitaxel (as a positive control) were, respectively, calculated at 6.666 ± 0.78 and 2.527 ± 0.37 µM using the MTT assay. Likewise, using the results of the MTT analysis for NFBTA and paclitaxel (at IC_50_), SI values were, respectively, calculated as 14.621 and 8.525. The results obtained from the cytotoxicity testing indicate that NFBTA presents good cytotoxic potential with a high SI value (>2.0) (Figure 3 and Figure 4; Table 1).

**Figure 5:**
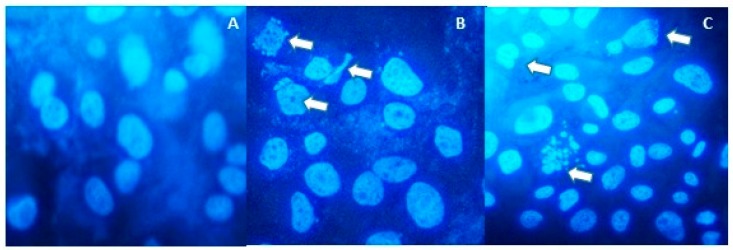
Cell apoptosis after treatments with paclitaxel and NFBTA for 48 h. Apoptosis was observed using Hoechst 33,258 staining. (**A**) Control cells without treatment, (**B**) cells treated with paclitaxel (at IC_50_; 2.53 μM), (**C**) cells treated with NFBTA (at IC_50_; 6.67 μM). Arrows indicate apoptotic bodies.

**Figure 6:**
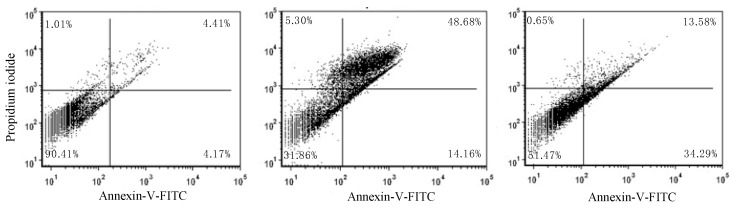
Effects of NFBTA on cell apoptosis. Flow cytometric analysis using Annexin V/propidium iodide staining to analyze apoptosis in U87MG cells. (Left) no treatment (control), (center) treatment with paclitaxel (at IC_50_; 2.53 μM), (right) treatment with NFBTA (at IC_50_; 6.67 μM) for 48 h.

**Figure 7:**
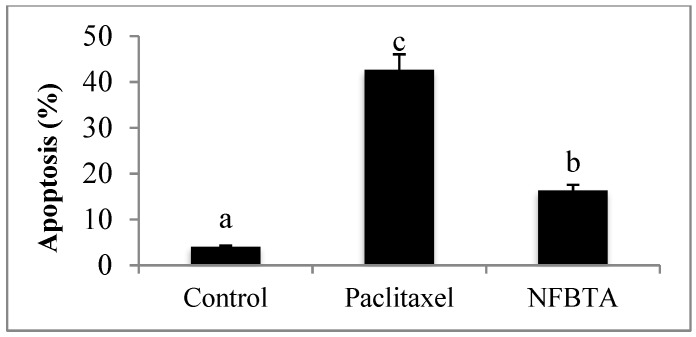
Apoptosis percentages after treatment with paclitaxel (at IC_50_; 2.53 μM) and NFBTA (at IC_50_; 6.67 μM) for 48 h. Different letters present statistically significant differences between groups, one-way ANOVA followed by Duncan’s test, of *p* < 0.05.

As shown in Figure 5A, no features of apoptosis were detected in cells without treatment. In Figure 5B, cells were treated with paclitaxel as a positive control, and apoptotic bodies were observed. In Figure 5C, cells were treated with the IC_50_ concentration of NFBTA, and apoptotic bodies with features of apoptosis were detected. Apoptosis was also confirmed by flow cytometry based on fluorescent staining of both treated and untreated cells. The percentage of apoptotic cells induced by NFBTA was determined by Annexin V and propidium iodide (PI) staining using cytometric flow analysis (Figure 6 and Figure 7). 

### 2.2. Oxidative Effects of NFBTA on U87MG Cells

**Figure 8:**
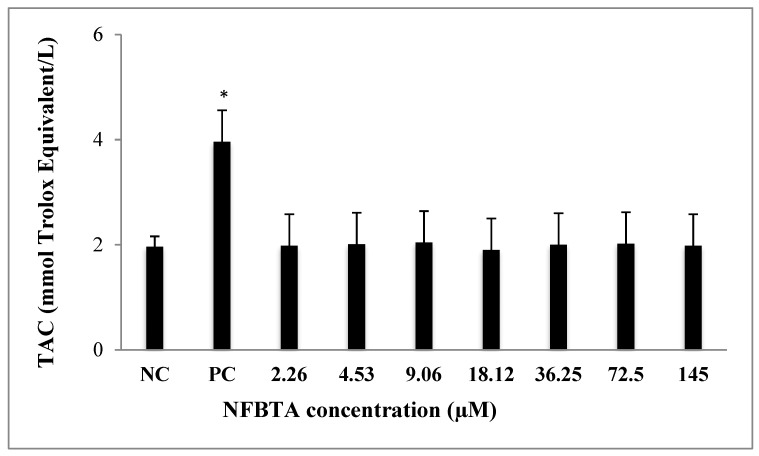
The effects of NFBTA applications (2.26–145 µM) on TAC levels in U87MG cells for 48 h. Data are expressed as the mean ± SD of four repetitive experiments. Statistical analysis was carried out by ANOVA and Duncan’s test. (*) symbol presents statistical differences between treated (NFBTA) and negative control (NC) cultures at a significance level of *p* < 0.05. PC: Positive control (ascorbic acid, 10 µM).

**Figure 9:**
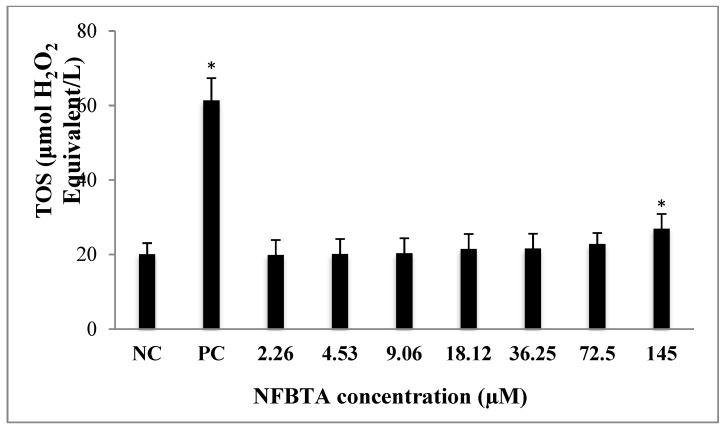
The levels of total oxidant status (TOS) in U87MG cells treated with different NFBTA concentrations (2.26–145 µM) for 48 h. Data are expressed as the mean ± SD of four repetitive experiments. Statistical analysis was carried out by ANOVA and Duncan’s test. (*) symbol presents statistical differences between treated (NFBTA) and untreated (NC) cultures at a significance level of *p* < 0.05. PC: Positive control (hydrogen peroxide, 25 µM).

Alterations of TAC and TOS levels were assessed in samples obtained from treated and untreated cell cultures via colorimetric measurement methods. As can be seen in Figure 8, applications with NFBTA (2.26 to 145 µM) led to insignificant increases in TAC levels for U87MG cells in comparison to the controls. Treatments with NFBTA (except for 145 µM) caused no significant alterations in TOS levels. Indeed, as compared to the untreated cultures, the highest concentration of NFBTA caused the TOS level to increase by 34.06% in the U87MG cell line (Figure 9).

### 2.3. Molecular Responses in U87MG Cells to NFBTA

**Figure 10:**
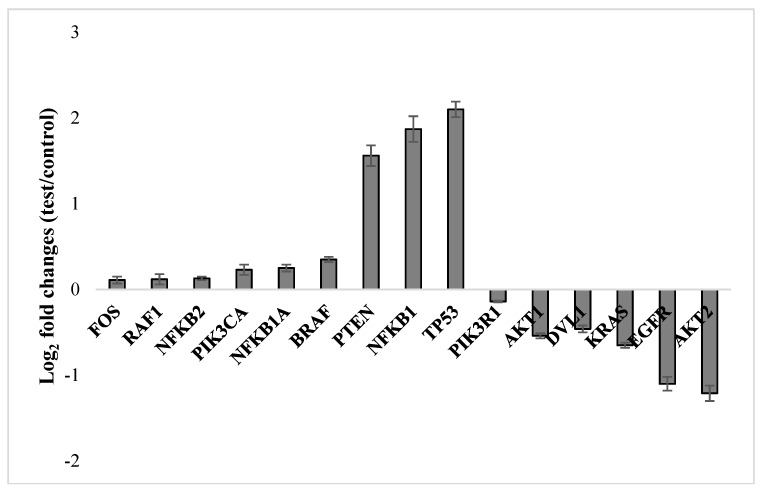
Gene expression alterations upon treatment with NFBTA (at IC_50_; 6.67 μM) in cultured U87MG cells for 48 h using 7500 Fast System SDS software. Data are expressed as the mean ± SD of four repetitive experiments. Fold change (y axis) represents the relative expression of the NFBTA mRNA in comparison to the control group normalized by the reference gene expression.

In order to determine whether NFBTA treatment changes certain gene expression levels, U87MG cells were treated with NFBTA (at the IC_50_ concentration of 6.67 µM) and 15 principal gene expression levels were measured via mini-microarray analysis. The molecular genetic response analysis performed revealed significant up- and down-regulation of the examined key genes related to carcinogenic pathways as compared to the untreated cultures (Figure 10). Thus, genes, including *FOS, RAF1, BRAF, NFKB1, NFKB1A, NFKB2, PIK3CA, PTEN,* and *TP53*, were up-regulated, while genes, including *AKT1, AKT2, DVL1, EGFR, KRAS*, and *PIK3R1*, were down-regulated.

### 2.4. Biosafety Assessments

**Figure 11:**
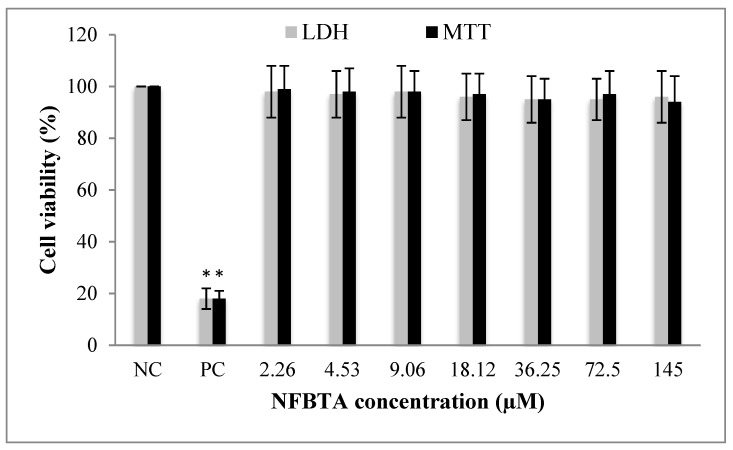
Cytotoxic effects of different NFBTA concentrations (2.26–145 µM) on human blood cell cultures for 48 h. Cell viability was determined using MTT and LDH assays. The values represent averages of four independent experiments with triplicate measurements (mean ± SD). Statistical analysis was carried out by ANOVA and Duncan’s test. (*) symbol presents statistical differences between treated (NFBTA) and untreated (NC) cultures at a significance level of *p* < 0.05.

MTT and LDH release assays were carried out to evaluate the in vitro biosafety profile of NFBTA. A wide range of NFBTA concentrations (from 2.26 to 145 µM) was applied to peripheral human whole blood (PHWB) cells. Treatment with all NFBTA concentrations presented no significant (*p* > 0.05) alterations in cell viability rates at 48 h, as measured via MTT reduction and LDH release analysis (Figure 11).

Table 2 presents oxidative alterations after treatment with different concentrations of NFBTA in PHWB cells for 48 h. The colorimetric measurement results for TAC and TOS revealed that, similar to U87MG cell cultures, NFBTA treatments led to significant oxidative alterations in cultured PHWB cells. The NFBTA treatments elevated TAC levels in a clear concentration-dependent manner. NFBTA concentrations higher than 4.53 µM supported the antioxidant capacity of the PHWB cells cultures between the rates of 22.54% and 95.30% without elevating the oxidant status.

**Figure 12:**
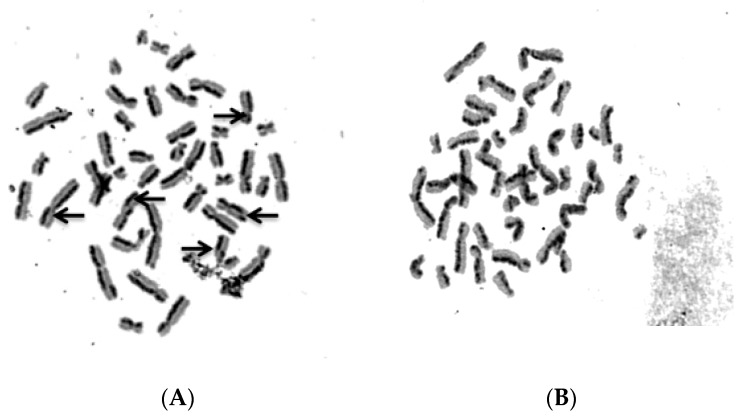
Sample metaphase micrographs from preparations after sister chromatid exchange (SCE) staining (**A**) Positive control, MMC (0.09 µM), and (**B**) NFBTA (145 µM) applications for 72 h (arrows indicate SCE formations).

To evaluate sister chromatid exchange (SCE) modulation with NFBTA treatment, SCE frequencies were scored in human lymphocyte cultures. Our analysis revealed no significant (*p* > 0.05) alterations in SCE rates (Table 3, Figure 12) for all NFBTA treatments. Table 3 also reflects the observed levels of 8-hydroxy-2′-deoxyguanosine (8-OH-dG) adducts in human blood cell cultures treated with NFBTA. It was determined that 8-OH-dG concentrations increased after mitomycin-C (MMC; as positive control) treatment at 72 h. At all tested NFBTA concentrations, no increase in 8-OH-dG adduct levels for cultured blood cells were observed.

## 3. Discussion

In the present study, to explore the potential anticancer activity of NFBTA, cytotoxic effects were determined using the MTT and LDH assays, apoptotic activity was determined using Hoechst 33,258 staining, DNA damage potential was determined using SCE and 8-OH-dG assays, and oxidative status was determined using the TAS and TOS methods. The molecular genetic response to NFBTA treatments was determined using an RT-PCR based quantitative assay. Our findings revealed that NFBTA presented strong anticancer potential against U87MG cells. In accordance with this finding, it was determined that PPL and its trimethoxy aromatic ring containing analogues were effective against human breast carcinoma MCF-7 cell proliferation [32]. The anticancer potential of PPL was also observed against HL-60 leukemia cells [33]. PPL has been reported to be effective against melanoma in both in vitro (B16F10-Nex cells) and in vivo (tumor bearing mice) experiments [34]. Values obtained from recent studies indicated that the antitumor activity of PPL was more prominent than its other pharmacological properties, such as anti-diabetic, anti-depressant, antiplatelet aggregation, and antifungal activities, as demonstrated through H-bonds and other affinity parameters [35].

NFBTA application did not promote the antioxidant capacity in U87MG cells, but in fact, NFBTA (145 µM) led to the generation of oxidative stress. In parallel with these results, it has been noted that PPL causes apoptotic cell death by promoting increased intracellular ROS levels in various human cancer cells lines, including in glioblastoma (LN 229, U87, and 8MG; IC_50_ = 20 µM), in addition to inhibiting the migration of the cancer cells, LN 229 and U87 [12,18], ovarian cancer (A2780, OVCAR3, and SKOV3; IC_50_ = 6–8 µM) [36], myeloid leukemia (bone marrow mononuclear cells; IC_50_ < 20 µM) [13], melanoma (MDA-MB-435; IC_50_ = 7 µM), and colon cancer (HCT-8; IC_50_ = 2.2 µM and HT-29; IC_50_ = 1.4 µM) [37,38]. NFBTA (IC_50_ = 6.666 ± 0.78 μM) was more potent than piplartine tested against glioblastoma cells when compared to previous studies (IC_50_ = 20 μM). It appears to be equipotent or less potent against some types of tumor cells. However, the synthesis of this compound in a single step indicates the lowest cost in the production and represents a great advantage. Interestingly, after application of the highest NFBTA concentration (145 µM), the antioxidant capacity of human blood cultures presented no elevations in oxidant levels. Addressing this point, it is first concluded that NFBTA causes oxidative stress in glioblastoma cells, but not in human blood cells. These findings indicate a selective inducement of oxidative stress (by PPL) in cancerous and normal cells, which may be a key mechanism underlying its antitumor action. However, the exact molecular mechanisms of this anticancer activity are still unclear. Due to the imperfection of reports on this important parameter, we performed detailed NFBTA molecular genetic response studies in U87MG cells.

Molecular analysis revealed that NFBTA significantly up-regulated genes, including *FOS, RAF1, BRAF, NFKB1, NFKB1A, NFKB2, PIK3CA, PTEN,* and *TP53*. Recently, various investigations have established that changes in gene expressions are directly associated with cancer, as well as the prevention of cancer. Distinct from the previously reported strongly cytotoxic PPL analogue (compound 10), *FOS* and *RAF1* were up-regulated by NFBTA [23]. The observed *FOS* gene expressional differences found between NFBTA and compound 10 have provided new insight into our novel PPL analogue, NFBTA. Previous reports revealed that *FOS* exhibited tumor suppressor activity and played significant roles in apoptosis [39]. The up-regulation of *FOS* inhibited cell cycle progression, induced cell death, and suppressed tumor formation [40]. Proto-oncogene serine/threonine-protein kinase (RAF) involvement in glioma genesis remains to be studied [41]. Recently, it was reported that *BRAF* (which encodes a serine/threonine protein kinase) mutation was closely related to improved survival in glioblastoma [42]. The observed up-regulation of *BRAF* expression by NFBTA is thus considered critical since *BRAF* is known to activate apoptosis stimulated by mitogen-activated protein kinase (MAPK or MAP kinase) [43], and MAPK signaling pathways in glioblastoma cells [42]. A special family of transcription factors named necrosis factor kappa B (NF-ĸB) plays critical roles in cancer initiation and progression [44], and mRNA expression of nuclear factor kappa B subunit 1 (NF-ĸB1) has been shown to be down-regulated in multiple hematological malignancies [45,46]. Recently, NF-kB1 was identified as an effective suppressor in inflammation, ageing, and cancer [47]. Yet, conversely, the constitutive activation of NF-κB has been found to be supportive in the growth and survival of glioblastoma cells [48], for the induction of anti-apoptotic gene expression [49]. Within this context, alterations of NF-κB pathways in GBM present a remarkable scientific challenge [50]. Indeed, the genes related to NF-κB-regulation exert key roles in modulating ROS within the cell, and at the same time, ROS are reported to exhibit both inhibitory and stimulatory effects on NF-κB signaling [46]. Thus, the exact function of the NF-κB pathway or its targeting of related transcription factors in glioma genesis remains undetermined. Curiously, it has been demonstrated that NF-κB activation does not always provide cellular apoptosis protection to glioma cells, and in addition, NF-κB activation together with the activation of tumor protein p53 (TP53) causes cell death [49]. Contrasting mechanisms have emerged based on both the cancer and cell type. Thereof, more detailed research is required to gain better insight into the exact role of *NFKB1* in glioblastoma. Mutations in the *PIK3CA* gene promote tumorigenesis [41]. Our results (after treatment with NFBTA in U87MG cells) also reflect significant elevations involving two principal tumor suppressor genes, phosphatase and tensin homolog (*PTEN)* and *TP53*. A previous study has suggested that the activation of *PTEN* induces p53-mediated cell cycle arrest [51]. Activated *PTEN* therefore promotes apoptotic cell death and the up-regulation of p53 expression in human hepatoma HepG2 cells [52]. This *PTEN* and p53 complex has been reported in association with cell cycle arrest, thus modulating DNA binding and transcriptional p53 activity [53].

Our results also revealed that NFBTA down-regulated the genes containing *AKT1, AKT2, DVL1, EGFR, KRAS*, and *PIK3R1*. Increased levels of serine/threonine kinases (*AKT*s) were detected in about 80% of all GBM cases. Moreover, *AKT2* expression has been found to roughly correlate with GBM progression and causes reductions in patient survival [54,55,56,57]. A very recent study has revealed that activation of the PI3K/AKT pathway is associated with the promotion of glioma stem cells’ (GSCs) self-renewal and tumor formation [58]. NFBTA may be proposed as a promising compound with therapeutic potential since inhibition of the PI3K/AKT/mTOR pathway has been suggested as a rational strategy for targeting GSCs. In accordance with our findings, epidermal growth factor receptor (EGFR) is overexpressed in nearly 60% of primary glioblastoma cases. In glioblastoma, this overexpression leads to increases in cell survival, proliferation, and invasion [59]. Similarly, genetic up-regulation of disheveled segment polarity protein 1 (*DVL1*) contributes to human glioma proliferation and invasion [60,61]. Increased expressions of *DVL1* have been strongly correlated with pathological grades of glioma [62]. Down-regulation of the genes mentioned, through treatment with NFBTA in U87MG cells, suggests that this new analogue is promising as a multi-action agent against glioblastoma. Further, NFBTA down-regulation of phosphoinositide-3-kinase regulatory subunit 1 *(PIK3R1)* genes augments its anti-glioblastoma efficacy, since knockout or silencing of *PIK3R1* genes evokes the responses of decreased proliferation, migration, and invasion in 081,110, and 081,024 GBM cell lines [63].

The computational target fishing approach provided a total of 91 potential targets for NBFTA. The targets with the highest values of consensus scores were: MMP1, TNR1A, MMP2, TBB1, LGUL, MMP9, transient receptor potential cation channel subfamily V member 1 (TRPV1), and HDAC1. It must be noted that target fishing methods rely on molecular similarity, hence not all potential targets can be provided by this type of method and not all predicted targets can explain the experimental results. In this sense, an expert criterion is required to select the potential targets for NBFTA. Taking this into consideration, the MMP-2, MMP-9, 90 kDa heat shock protein (HSP90), and TRPV1 targets were employed for further investigation.

The above described docking consensus scoring procedure for the selection of the best ligand conformer was validated using the experimentally determined ligands structures for MMP-2, MMP-9, and HSP90. The results of this validation are presented in Table 4 and it must be highlighted that these calculations were aimed at reproducing the experimental binding mode of each compound from their planar structures. The low root-mean-square deviation (RMSD) values (<2 Å) relative to the experimental binding modes obtained support the use of our consensus scoring protocol for the selection of close to experimental binding modes for these targets.

**Figure 13:**
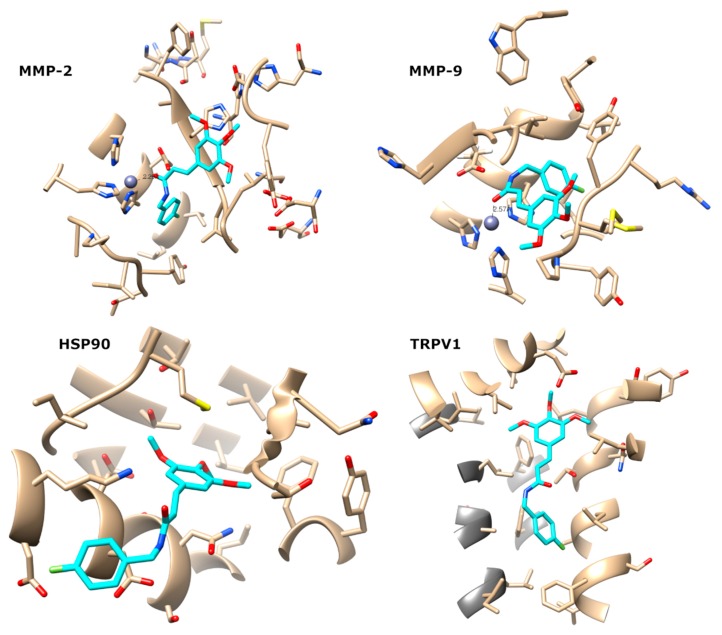
Predicted binding modes of NBFTA to MMP-2, MMP-9, HSP90, and TRPV1.

This consensus scoring protocol was further applied to the docking of NBFTA to MMP-2, MMP-9, HSP90, and TRPV1. The fitness and Z-scores obtained for NBFTA are summarized in Table 5 and it is observed that the predicted scores for NBFTA are lower than those obtained for the reference ligands used in the consensus scoring protocol validation. This behavior is expected since the compounds used for the protocol validation are all high affinity ligands of their respective targets. To gain further insights into the predicted complexes of NBFTA with the four targets, we examined the predicted binding poses as shown in Figure 13.

The predicted complexes of NBFTA to the four targets show extensive contact with all of them. In the case of metalloenzymes 2 and 9, there is a clear interaction between the carbonyl group of NBFTA with the Zn^2+^ atom present at the active site of the enzymes. In both cases, the O-Zn^2+^ distances are lower than 2.6 Å. The complex, NBFTA/MMP2, also shows a large number of ligand–receptor contacts. For example, the fluorophenyl group of NBFTA makes an extensive network of interactions with LEU83, VAL117, GLU121, TYR142, and HIS120, while positions favorably for π- π stacking with the latter. Furthermore, the trimethoxyphenyl moiety of NBTFA engages in a network of contacts with TYR74, ASP72, LEU 82, and HIS85 of MMP-2.

Besides a hydrogen bond between the NH group of NBFTA and the side chain of GLU122, the predicted NBFTA/MMP-9 complex shows extensive contact of the ligand with LEU83, MET142, PRO141, and TYR143 through its trimethoxyphenyl group. Additional contact is also observed with VAL118, HIS121, GLY122, and TYR140. In the case of the complex with HSP90, two hydrogen bonds are predicted with LYS43 and ASP39 while the fluorophenyl moiety of NBFTA points toward the solvent. The contact of the ligand with HSP90 include ASN36, ALA40, MET83, LEU92, LEU93, THR94, PHE123, THR169, and VAL171.

Finally, NBFTA is predicted to bind to the TRPV1 vanilloids site formed by a large hydrophobic channel located between two subunits in the homo-tetramer. As for MMP-2, no hydrogen bond is predicted between NBFTA and TRPV1. However, the compound makes an extensive network of hydrophobic contact with TRPV1 that includes LEU515, THR550, ASN551, LEU553, GLU569, and ILE573 located at helices S3 and S4 and at the S4–S5 linker [64]. Moreover, contact with PHE560, LEU670, LEU674, PHE591, and ALA666 from the adjacent monomer is also predicted.

Most of the above described interactions have been observed for other ligands of these targets. At the same time, all predicted complexes present interactions that support the formation of stable complexes. Furthermore, the obtained docking scores show little discriminant ability between targets. Taken together, the molecular docking results are unable to discriminate between the four targets being investigated for NBFTA. To gain further insights into the possible mechanism of action of this compound, we performed molecular dynamics (MD) simulations and the free energy of binding calculations of the previously predicted complexes.

**Figure 14:**
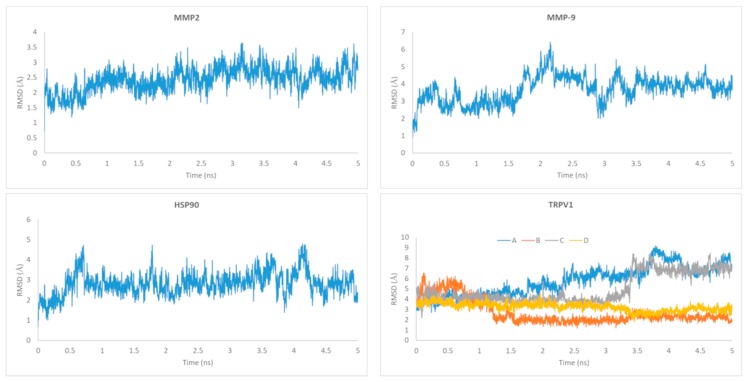
Ligand root mean square deviation (RMSD) relative to the initial docking structure. For TRPV1, the corresponding RMSD values for each of the ligands present in the four binding pockets are represented.

The RMSD of the ligand relative to the starting docking structure was evaluated and is summarized in Figure 14. As can be observed, there are fluctuations of the RMSD value for all ligands relative to their initial conformations, with the ligands docked to the vanilloids binding sites of the A and C subunits of TRPV1 being the most flexible ones. Despite this flexibility, all trajectories show stability at the last nanosecond that was selected for the molecular mechanics Poisson–Boltzmann/surface area (MM/PBSA) calculations. The receptors’ backbones also remained stable along the full MD simulations (data not shown).

As previously described, the last nanosecond of the MD simulations was selected for the free energy of binding calculations employing the MM/PBSA approach. The results of these calculations are summarized in Table 6. Despite the meaningful docking predictions obtained for the four targets, from these simulations, it is clear that favorable binding energy values for NBFTA are only obtained for TRPV1. The MM/PBSA approach, in contrast to molecular docking, takes into account the effect of the solvent in ligand binding. This is a major factor to consider when molecular binding processes are investigated. Thus, the positive (unfavorable) free energies observed for the complexes formed with MMP-2, MMP-9, and HSP90 could be explained from the fact that the ligand–receptor interactions predicted in these complexes are unable to compensate the energetic penalty associated with the ligand’s and receptors’ desolvation. In the specific case of the metalloenzymes, many inhibitors interact with the Zn^2+^ ion through two carbonyl groups. This leads us to speculate that the addition of one extra carbonyl group to NBFTA close to the one already present could make it a possible metalloenzyme inhibitor. It has also been previously demonstrated that the simultaneous presence of ligands in the four vanilloids binding sites can improve their agonist activity. Our calculations are in agreement with this observation since favorable binding in all four binding sites was observed and the free energy of binding of the four NBFTA molecules to TRPV1 equals to −165.63 ± 5.5 kcal/mol.

There are literature reports linking the activation of TRPV1 to the anticancer effect of chemical compounds [65,66,67]. The mechanism of TRPV1-mediated cytotoxicity involves cellular apoptosis through the accumulation of Ca^2+^ in the mitochondria [66,68]. Specifically, glioblastoma cell lines have been demonstrated to be sensitive to TRPV1 agonists and to overexpress this receptor [69,70,71]. All these experimental evidences, along with the modeling results, support our proposal that the anti-proliferative activity of NBFTA relates to the activation of the TRPV1 receptor.

Although the structural mechanism by which TRPV1 is activated is not yet fully understood, the interaction between ARG557 and GLU570 has been shown to be essential for its agonist-mediated activation [72]. This interaction was confirmed in the structure of TRPV1 in the complex with the agonist, resiniferatoxin, where the compound forms a hydrogen bond with ARG557 and the latter interacts through a hydrogen bond with GLU570. In this structure, the agonist molecule stabilizes the interaction between the two residues, which leads to the conformational changes required for TRPV1 activation.

**Figure 15:**
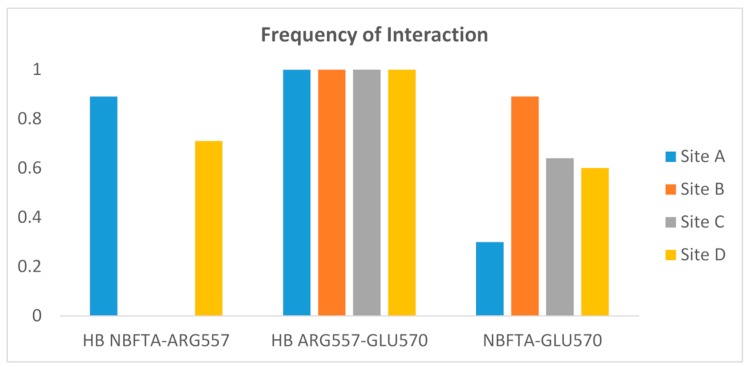
Frequency of interaction between NBFTA, ARG557, and GLU570 during the last nanosecond of the NBFTA–TRPV1 molecular dynamics (MD) simulation. Separate calculations were performed per vanilloids binding site. HB indicates hydrogen bonds while NBFTA–GLU570 indicates non-hydrogen bond interactions between these residues.

The above interactions were not observed in the docking model of the NBFTA–TRPV1 complex due to the initial receptor conformation. To explore whether these interactions took place during the MD simulation of the NBFTA–TRPV1 complex, we monitored their presence during the last nanosecond used for the MM/PBSA calculations. The results of this analysis are summarized in Figure 15 and show that the hydrogen bond between ARG557 and GLU570 is permanently formed in the four vanilloids binding sites. Thus, NBFTA can indeed have an agonist effect on TRPV1. Furthermore, the ligand molecules present in the binding sites of subunits A and D of TRPV1 interact with ARG557 through a hydrogen bond in 89% of the 500 MD snapshots included in the last nanosecond of simulation. This last observation is in agreement with the agonist mechanism previously described for TRPV1 agonists. Finally, the NBFTA–ARG557 hydrogen bond interaction was not observed for the ligands bound to subunits B and C of the receptor. However, visual inspection of the MD trajectory shows that in these cases, NBFTA interacts with GLU570 and orients it toward ARG557, favoring the formation of the ARG557–GLU570 hydrogen bond.

**Figure 16:**
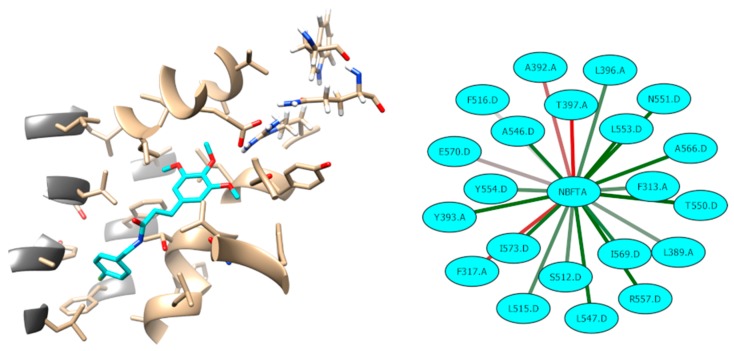
Interactions between NBFTA and TRPV1 (subunit D). Interaction frequency goes from red (low) to green (high). Residues from the A subunit are shown with dark gray ribbons.

Finally, we analyzed the network of interactions between NBFTA and TRPV1 at the binding site of subunit D. We also clustered the NBFTA conformations during the last nanosecond of the MD simulation for this binding site. These analyses are summarized in Figure 16, where it can be seen that most NBFTA–TRPV1 interactions, in addition to the previously described ones, form a large network of a hydrophobic nature. Furthermore, NBFTA occupies the well-known vanilloids site, which also contains residues from the adjacent subunit, at the same time that its orientation resembles that observed for other TRPV1 agonists. In summary, our results strongly suggest a plausible anti-proliferative mechanism for NBFTA and this study could serve for the design of additional experiments to further confirm our hypothesis.

## 4. Materials and Methods

### 4.1. Preparation of NFBTA

3,4,5-Trimethoxycinnamic acid (0.42 mmol) was dissolved in 0.84 mL of dimethylformamide (DMF) and 0.06 mL (0.42 mmol) of triethylamine. The solution was cooled in an ice water bath, and 0.42 mmol of 4-fluorbenzylamine was added, followed by a 0.42 mmol solution of benzotriazole-1-yl-oxy-tris-(dimethylamino)-phosphonium hexafluorophosphate (BOP) in 0.84 mL of CH_2_Cl_2_. The mixture was stirred at 0 °C for 30 min, and then at room temperature for 3 h. Afterwards, the CH_2_Cl_2_ was removed under reduced pressure, and the solution diluted with 10 mL of water. The products were extracted three times with 10 mL of ethyl acetate. The product was successively washed with 1N HCl, a solution of 5% (w/v) NaHCO_3_, in water, dried over anhydrous Na_2_SO_4_, and filtered. The solvent was removed under vacuum. The product was then isolated using silica gel column chromatography (mobile phase, ethyl acetate/n-hexane 4:6, v/v). A white solid was obtained (MM: 345.14 g/mol, Mp: 195–196 °C, 0.1260 g, yield 87%). ^1^H NMR (200 MHz, CDCl_3_) δ 7.55 (d, *J* = 16.0 Hz, 1H), 7.25 (m, 2H), 6.97 (t, *J* = 8.0 Hz, 2H), 6.68 (s, 2H), 6.37 (d, *J* = 14.0 Hz, 1H), 4.48 (d, *J* = 6.0 Hz, 2H), 3.84 (s, 3H), 3.81 (s, 6H), ^13^C NMR (50 MHz, CDCl_3_) δ 166.0, 164.7, 159.8, 153.4, 141.5, 139.6, 134.1, 130.4, 129.6, 129.5, 119.8, 115.8, 115.4, 105.0, 61.0, 56.1, 43.2. IR v_max_ (KBr, cm^−1^) 3291, 3012, 2959, 2932, 2839, 1651, 1616, 1580, 1508, 829, 613 [73,74].

### 4.2. Cell Cultures and Treatments

The U87MG cell line was obtained from the American Type Culture Collection, (Rockville, MD, USA). The cells were cultivated in Eagle’s Minimal Essential Medium (EMEM) enriched with 10% heat inactivated fetal bovine serum (FBS), 100 U/mL of penicillin, and 100 μg/mL of streptomycin at 37 °C in a CO_2_ incubator. Human pulmonary alveolar epithelial (HPAEpiC) cells were obtained from ScienCell Research Laboratories (Carlsbad, CA, USA), and cultured according to the manufacturer’s instructions in alveolar epithelial cell medium supplemented with growth factors. NFBTA was dissolved in DMSO and diluted with medium prior to use. The final concentration of DMSO in the cultures was adjusted to <0.1% (v/v). A control experiment was also carried out by replacing NFBTA with the same amount of DMSO (to manage any possible effects of DMSO on the cell growth). Triton X-100 (%1) and Mitomycin-C (0.09 µM) were added to the cells as positive control agents for testing of the cytotoxicity and genotoxicity [60,75].

### 4.3. MTT Assay

Cells were plated to each well of 48-well plates (1 × 10^5^ cells/well). After 24 h, the plated cells were treated with a dosage of NFBTA (2.26, 4.53, 9.06, 18.12, 36.25, 72.5, or 145 µM) for 48 h and placed in a 5% CO_2_ incubator at 37 °C. Upon completion of the incubation period, the MTT solution (Gibco-BRL, USA) was added at a 1:10 ratio and again kept in the CO_2_ incubation chamber for 3 h. After 3 h of incubation, the media was removed, and formazan (dissolved by DMSO) (Sigma, USA) was added to each well. The optical density of each sample was measured with a plate reader at a wavelength of 595 nm [60,76]. The IC_50_ values were defined using the MTT assay results. The selectivity index (SI) was then calculated using the IC_50_ value for normal lung cells (HPAEpiC) as divided by the IC_50_ value of the U87MG cells also obtained from MTT analysis.

### 4.4. LDH Assay

The LDH level was evaluated using a commercial kit (Cayman Chemical Company^®^, Ann Arbor, MI, USA) according to the manufacturer’s instructions. Briefly, 100 µL of cell supernatant treated with a dosage of NFBTA (2.26, 4.53, 9.06, 18.12, 36.25, 72.5, or 145 µM) was moved to a new 48-well plate and mixed with 100 µL of assay solution. After 30 min of incubation, optical density was determined at 490 nm using a plate reader [60,76].

### 4.5. Total Antioxidant Capacity (TAC) and Total Oxidant Status (TOS) Assays

TAS and TOS levels were measured in treated and untreated cell cultures using assay kits (Rel Assay Diagnostics^®^, Turkey) according to the manufacturer’s instructions.

### 4.6. Hoechst 33,258 Staining for Apoptosis Determination

Cells were first treated with a fixative (4% paraformaldehyde) for 30 min at 4 °C, and then stained with Hoechst 33,258 for 5 min without light. The stained cells were then observed using a fluorescent microscope (Leica^®^ DM IL LED).

### 4.7. Annexin V-FITC/PI Apoptosis Assay

Apoptosis rates were determined using an Annexin V-FITC apoptosis detection kit I (BD Pharmingen). Briefly, 5 × 10^4^ cells were suspended in binding buffer. Annexin V-FITC and propidium iodide (PI, 50 μL) were then added to the cultures and incubated for 5 min. Following fixation with paraformaldehyde, the cells were stained in accordance with the manufacturer’s instructions and analyzed via a flow cytometer (CyFlow Cube 6, Partec^®^).

### 4.8. qRT-PCR Assay

The qRT-PCR assay was carried out as described previously [45]. Briefly, cells were treated with NFBTA at the IC_50_ dosage for 48 h. For the RNA extraction process, cell cultures were harvested and gathered in a single tube and the PureLink^®^ RNA Mini Kit (Invitrogen, MA, USA) was used to isolate the total RNA according to the provider’s instructions. For cDNA synthesis, RNA was used with a High-Capacity cDNA Reverse Transcription kit (Applied Biosystems). PCR assay was then performed using the TaqMan^®^ Custom Assay (Applied Biosystems) for the monitoring of mRNA expression in the targeted genes (*EGFR*, *AKT1*, *AKT2*, *NFKB1*, *NFKB1A*, *NFKB2*, *PTEN*, *KRAS*, *PIK3CA*, *PIK3R1*, *TP53*, *RAF1*, *BRAF*, *DVL1*, *FOS*). Analysis procedures were carried out using the 7500 Fast System SDS software (Applied Biosystems) [60].

### 4.9. Biosafety Evaluation

Human peripheral blood cell cultures were used to evaluate NFBTA’s biosafety. The blood cultures were established according to Evans and O’Riordan’s (1975) procedures (with minor modifications). Whole blood was taken ethically by venipuncture using heparinized tubes from four healthy, male donors, aged 27 to 29 years, with no history of tobacco use, and no known previous exposure to mutagens. A 0.6 mL volume of peripheral blood was added and cultured in a tube containing (PB-MAX Karyotyping Medium Gibco, Spain) medium enriched with phytohemagglutinin (5.0 mg/mL, Sigma Aldrich, Steinheim, Germany). The blood cells were exposed to several NFBTA concentrations. MTT and LDH assays were used to determine NFBTA cytotoxicity. TAC and TOS assays were performed to detect oxidative alterations. Sister chromatid exchange (SCE) and 8-hydroxy-2′-deoxyguanosine tests were used to assess genotoxicity [60].

### 4.10. SCE Testing

To visualize the SCEs, 5-bromo-2-deoxyuridine (Sigma Aldrich ^®^) was added to the cultures at the beginning of incubation. Demecolcine (N-Diacetyl-N-methylcolchicine, Sigma Aldrich ^®^) was added to the cultures after 70 h and 35 min of culture initiation. After completing 72 h, the cells were collected in a tube by centrifugation and treated with KCl (0.075 M) as a hypotonic solution. The cells were then fixed three times in cold methanol/acetic acid solution (3:1, v/v). The cell suspension was dropped onto cold slides and kept overnight. Air-dried slides were stained according to the Fluorescence Plus Giemsa (FPG) technique. For the scoring of SCEs, well-spread 25 metaphases containing 42 to 46 chromosomes per cell were counted, and the values were expressed as SCEs per cell.

### 4.11. Nucleic Acid Oxidation

8-OH-dG levels in the cultures were measured using an 8-hydroxy-2′-deoxyguanosine assay kit (Cayman Chemical Company^®^, Ann Arbor, MI, USA). All steps were performed in accordance with the manufacturer’s instructions.

### 4.12. Statistical Analysis

Data are presented as the means ± SD of at least four experiments. The quantitative data was analyzed by SPSS software (version 20.0, SPSS, Chicago, IL, USA). Duncan’s test and one-way analysis of variance (ANOVA) were performed to reveal statistical differences. The differences were considered as statistically significant with *p* < 0.05.

### 4.13. General Modeling Workflow

The first step of the computational modeling workflow was to identify potential targets for the anticancer activity of NBFTA by employing a consensus target fishing strategy. From the identified potential targets, a subset of them was selected as being the most probable for NBFTA. The compound was docked to each protein in this subset to obtain its possible binding modes. The predicted target–NBFTA complexes were then the subject of molecular dynamics (MD) simulations and free energy calculations. The latter were performed from the obtained MD trajectories. Unless otherwise noted, default parameters were employed in the simulations described below. Figures containing molecular structures were generated with UCSF Chimera [77] and network interactions were visualized with Cytoscape [78].

### 4.14. Computational Target Fishing

The in-silico prediction of targets for the compound of interest was done by combining three target-fishing methods: MolTarPred [79], PredictionCharite [80], and similarity ensemble approach (SEA) [81]. Each of these methods retrieves a list of potential targets as well as a score or ranking. In order to integrate the three methods, we followed a fusion scoring strategy previously used for gene prioritization [82,83]. In brief, this strategy consists of:

(1) Determine the ranking of all targets according to each method and define $R_{i}^{j}$ as the normalized ranking of the target, *i*, according to the method, *j*.

(2) Compute how many times the target, *i*, is predicted across the three methods ($F_{i}$). This frequency can take values between 1 and 3.

(3) The final score of a target ($S_{i}$) is then calculated as: $S_{i}=\sqrt{\left(\frac{F_{i}}{3}\right)\left(\frac{\sum_{j}R_{i}^{j}}{3}\right)}$.

This equation represents the geometrical mean between the average of the normalized scores and the frequency of the appearance of the target. Therefore, a target would be more relevant if it is predicted by all the methods and also with a high score by each of them.

### 4.15. Molecular Docking

The structures of MMP-2 in complex with the SC-74020 inhibitor (PDB 1HOV), MMP-9 in complex with the CC27 inhibitor (PDB 4H3X), and the complex between the N-terminal domain of HSP90 with 4-chloro-6-(4-piperazin-1-yl-1h-pyrazol-3-yl)-benzene-1,2-diol (PDB 2CCS) were selected for modeling studies. For the human TRPV1 receptor, the homology model deposited at the Swiss-Model server [84] (https://swissmodel.expasy.org/repository/5bbb3794c4494f706edbe05c.pdb) was employed. All targets, except TRPV1, contained bound ligands that were used for the validation of the docking protocol. Molecular docking calculations were performed with the Gold software [85].

Experimental ligands were removed from the MMP-2, MMP-9, and HSP90 targets and represented as planar structures. The most stable conformation of each ligand was obtained by means of the OMEGA software [86]. AM1-BCC charges were added to the ligands with MOLCHARGE [87]. The same procedure was used to generate the initial 3D conformation of NBFTA.

For docking, all water molecules and crystallization co-factors were removed from targets. Zinc atoms were kept on MMP-2 and MMP-9. The binding sites of the proteins were defined as a region within 6 Å from the bound reference ligands. For TRPV1, the receptor model was superimposed to the rat TRPV1 structure with resiniferatoxin bound at the vanilloids binding site (PDB 5IRX) and the latter was used as the reference ligand.

In total, 30 different possible binding modes of the ligands were explored with GOLD. Piecewise linear potential (CHEMPLP) was used as the primary scoring function and the flexibility option was set to very flexible (200% search efficiency). The predicted binding modes were then rescored with the GoldScore, ChemScore, and ASP scoring functions.

A consensus scoring protocol was employed to select the best conformer of the ligands. This approach consisted in scaling the score of conformer, *C_i_*, according to the scoring function, *S_j_* (*S_i,j_*), as:

(1) $$Z_{i,j}=\frac{S_{i,j}-\bar{S_{j}}}{std\left(S_{j}\right)},$$

where $\bar{S_{j}}$ is the mean of the scoring function, *S_j_*, across all conformers, and $std\left(S_{j}\right)$ is the standard deviation of the *S_j_* values. Next, the scaled scores of each conformer, *C_i_*, were summed to obtain the consensus score, *Z_i_*, and the most probable binding mode of the compound was selected as the conformer with the largest *Z* score. This docking protocol was first validated using the experimental reference ligands and then applied to NBFTA.

### 4.16. Molecular Dynamics Simulations

Molecular dynamics (MD) simulations were performed with Gromacs 5.1.4 [88]. The most probable complexes of NBFTA with each receptor identified from the docking studies were used as the initial structures for MD simulations. MMP-2, MMP-9, and HSP90 systems were modeled in explicit solvent while TRPV1 MD simulations were performed in an explicit membrane environment as described below. All MD simulations were performed with the CHARMM36 force field and NBFTA topology was generated with the CGenFF 4.0 server [89].

For explicit solvent simulations, a dodecahedron box extending 10 Å from the complex was constructed. The system was solvated with SPC (spc216) water molecules and excess charges were neutralized by the addiction of either Na^+^ or Cl^−^ ions. In the case of TRPV1, only the transmembrane segments containing the vanilloids binding sites were considered for MD simulations. This system was modeled as a homo-tetramer containing one ligand per binding site. The complex was embedded in a 1-Palmitoyl-2-oleoylphosphatidylcholine (POPC) bilayer using the membrane builder module of the CHARMM-GUI server [90,91]. This system was also solvated and neutralized using the same server, thus obtaining an MD simulations ready setup.

Then, the system was energy minimized for a maximum of 50,000 cycles of steepest descent minimization using all directions’ periodic boundary conditions. Energy minimization stopped when the maximum force reached a value lower than 1000 KJ.mol^−1^.nm^−1^. The neighbors list was updated at every step using a Verlet scheme. Long range electrostatic interactions were treated according to the PME method with a cutoff of 1.2 nm. Vdwtype for energy minimization was set to a cutoff (1.2 nm) with a force-switch modifier, an rvdw-switch of 1 nm, and no corrections were applied.

Afterward, a 100 ps equilibration simulation with the protein and ligand restrained was carried out by employing the leap frog integration algorithm with a 2 fs time step. The LINCS algorithm was employed for the constraint of all bonds containing hydrogens. The neighbors list for the Verlet scheme was updated every 20 steps. Long range electrostatic interactions were treated as in the energy minimization step with a Fourier spacing of 0.16 nm. Temperature was controlled using a modified v-rescale Berendsen thermostat with the receptor and ligand in one group and solvent and ions in a second one. A third group containing the phosphatidylcholine (POPC) bilayer membrane was considered for temperature coupling in TRPV1. The time constant for coupling was set to 0.1 ps and the temperature was set to 300 K. Since this equilibration step was run in isothermal-isochoric (NVT) conditions, no pressure coupling was used. The equilibration was run with periodic boundary conditions and initial velocities were assigned according to a Maxwell distribution at 300 K.

Additional 100 ps equilibration with both the receptor and ligand constrained was performed for 100 ps in NPT conditions. In addition to the parameters employed in the previous equilibration step, pressure coupling was added. A Berendsen isotropic coupling was elected with a time constant of 2 ps and pressure set to 1 bar. In addition, the center of mass of the reference coordinates was scaled with the scaling matrix of the pressure coupling. Following equilibration, the production MD run was performed for 5 ns. Coordinates were saved every 2 ps. In contrast to the last equilibrium phase, a Parrinello–Rahman pressure coupling was selected for the production run.

### 4.17. MM-PBSA Calculations

MM-PBSA calculations to estimate the free binding energy of the complexes were performed with AmberTools 18 [92]. For this, 100 snapshots covering the last nanosecond (one every 10 ps) of the MD simulations were selected. The ionic strength was set to 150 mM for all systems. Default implicit solvent parameters were set for the MMP-2, MMP-9, and HSP-90 systems.

On the other hand, for TRPV1, an implicit membrane model recently introduced was employed [93,94]. For the implicit membrane simulations, it was set as a 40 Å solid slab covering the transmembrane section of TRPV1. Sander was set as the PB equation solver with dielectric factors of 20 and 7 for the exterior and membrane, respectively. The grid to solute dimension ratio was set to 1.25 and periodic boundary conditions were used.

## 5. Conclusions

In summary, the present study clearly described the potential anticancer activity of NFBTA through a set of experiments revealing its cytotoxic profile. NFBTA exhibited inhibitory effects against human GBM cell viability. Its molecular anticancer action mechanisms were principally associated with the BRAF/MAPK and PI3K/AKT signaling pathways. In addition, oxidative alterations and cell cycle arrest were also thought to be responsible for the in vitro cytotoxic potency of NFBTA. Molecular docking studies confirmed the interaction of this compound with biological targets related to antitumor potential in glioblastoma. The present findings demonstrated that NFBTA might provide leads to potential agents for glioblastoma therapy, and this potent new compound could itself be considered a promising cytotoxic agent for further studies against brain cancers.

## Figures and Tables

**Table 1:** Cytotoxic activity of NFBTA and paclitaxel; (IC_50_, μM) and SI values for U87MG and HPAEpiC cells.

Compounds	U87MG Cells	HPAEpiC Cells	SI Value
NFBTA	6.666 ± 0.78	97.385 ± 3.83	14.621
Paclitaxel	2.527 ± 0.37	21.545 ± 1.46	8.525

**Table 2:** NFBTA caused oxidative alterations in peripheral human whole blood (PHWB) cell cultures (n = 4) for 48 h.

Concentrations (µM)	TAC (mmol Trolox Equiv./L)	TOS (μmol H_2_O_2_ Equiv./L)
NC	4.3 ± 0.4	7.1 ± 0.8
PC	11.8 ± 0.7*	28.3 ± 1.6*
2.26	4.5 ± 0.4	6.9 ± 0.4
4.53	4.5 ± 0.6	6.9 ± 0.5
9.06	5.2 ± 0.6*	7.0 ± 0.6
18.12	5.5 ± 0.6*	6.9 ± 0.6
36.25	5.7 ± 0.5*	7.1 ± 0.5
72.5	7.1 ± 0.7*	7.1 ± 0.6
145	8.3 ± 0.7*	7.2 ± 0.6

PC: Positive controls (TAC assay, 10 µM ascorbic acid; TOS assay, 25 µM hydrogen peroxide). (*) symbol presents statistical differences between treated (NFBTA) and untreated (NC) cultures at a significance level of *p* < 0.05.

**Table 3:** Genotoxicity of different NFBTA concentrations on cultured human lymphocytes (n = 4) for 72 h.

Concentrations (µM)	SCEs/Cell	pmol 8-OH-dG/μg DNA
NC	5.9 ± 0.7	1.0 ± 0.2
PC	13.8 ± 1.8*	4.3 ± 0.3*
2.26	5.2 ± 0.6	1.0 ± 0.2
4.53	4.9 ± 0.5	1.1 ± 0.1
9.06	5.4 ± 0.6	1.1 ± 0.2
18.12	5.8 ± 0.4	0.9 ± 0.2
36.25	6.1 ± 0.7	1.1 ± 0.2
72.5	5.9 ± 0.6	1.2 ± 0.2
145	6.3 ± 0.8	1.1 ± 0.2

PC: Positive control (MMC: Mytomicin C, 0.09 µM). (*) symbol presents statistical differences between treated (NFBTA) and untreated (NC) cultures at a significance level of *p* < 0.05.

**Table 4:** Validation of the consensus scoring protocol.

	Target			
		MMP-2	MMP-9	HSP90
CHEMPLP	Fitness	99.18	101.71	58.22
	Z-score	2.71	1.33	2.54
GoldScore	Fitness	71.59	44.64	35.29
	Z-score	2.45	0.97	1.90
ChemScore	Fitness	43.01	30.36	21.71
	Z-score	2.73	1.72	2.62
ASP	Fitness	52.24	52.23	28.15
	Z-score	2.36	0.94	2.18
Consensus Z-score		10.25	4.97	9.24
RMSD (Å) ^(a)^		0.89	1.87	0.56

^(a)^ RMSD is computed relative to the experimental binding mode.

**Table 5:** Scores of the best predicted poses of NBFTA to the targets under investigation.

	Target				
		MMP-2	MMP-9	HSP90	TRPV1
CHEMPLP	Fitness	69.81	83.32	56.67	54.05
	Z-score	2.09	1.38	2.51	1.59
GoldScore	Fitness	31.34	38.04	27.28	25.44
	Z-score	0.57	0.02	0.82	0.57
ChemScore	Fitness	17.30	25.99	11.81	12.78
	Z-score	1.51	2.93	0.82	0.19
ASP	Fitness	33.92	35.29	19.82	28.71
	Z-score	1.53	0.69	−0.55	3.42
Aggregated Z-score		5.69	5.02	3.60	5.77

**Table 6:** Summary of the MM/PBSA calculations to estimate the free energy of binding of TRPV1 to the MMP-2, MMP-9, HSP90, and TRPV1 targets.

	MMP-2		MMP-9		HSP90		TRPV1									
							LIG A		LIG B		LIG C		LIG D		All Ligs	
Component	Average	Std.	Average	Std.	Average	Std.	Average	Std.	Average	Std.	Average	Std.	Average	Std.	Average	Std.
VDWAALS	−31.20	2.71	−30.75	3.45	−35.36	2.49	−37.48	3.29	−38.48	2.57	−26.69	1.95	−48.12	2.99	−150.77	5.55
EEL	−54.57	6.81	−54.33	6.22	−6.84	7.37	0.02	0.19	0.94	0.27	−0.12	0.33	1.07	0.22	1.90	0.59
EPB	73.54	9.17	66.93	5.29	34.23	7.73										
ENPOLAR	−23.86	1.24	−24.10	1.67	−27.04	1.64	−4.29	0.16	−4.48	0.09	−3.48	0.20	−4.51	0.12	−16.76	0.33
EDISPER	41.76	1.39	42.63	2.91	46.83	1.66										
ΔG gas	−85.77	7.47	−85.08	6.08	−42.19	7.48	−37.46	3.26	−37.54	2.58	−26.81	1.92	−47.05	2.98	−148.87	5.51
ΔG solv	91.43	9.25	85.46	5.78	54.01	7.59	−4.29	0.16	−4.48	0.09	−3.48	0.20	−4.51	0.12	−16.76	0.33
ΔG TOTAL	5.67	7.78	0.39	3.95	11.82	6.31	−41.76	3.27	−42.02	2.57	−30.29	2.03	−51.56	3.00	−165.63	5.55
